# A New Use for an Old Drug: Carmofur Attenuates Lipopolysaccharide (LPS)-Induced Acute Lung Injury *via* Inhibition of FAAH and NAAA Activities

**DOI:** 10.3389/fphar.2019.00818

**Published:** 2019-07-19

**Authors:** Kangni Wu, Yanghui Xiu, Pan Zhou, Yan Qiu, Yuhang Li

**Affiliations:** ^1^Department of Hematology, The First Affiliated Hospital of Xiamen University, Xiamen, China; ^2^Eye Institute & Affiliated Xiamen Eye Center, Xiamen University, Xiamen, China; ^3^Institute of Hematology, Medical College of Xiamem University, Xiamen, China; ^4^Xiamen Institute of Rare-earth Materials, Haixi Institutes, Chinese Academy of Sciences, Fujian, China; ^5^CAS Key Laboratory of Design and Assembly of Functional Nanostructures, and Fujian Provincial Key Laboratory of Nanomaterials, Fujian Institute of Research on the Structure of Matter, Chinese Academy of Sciences, Fujian, China

**Keywords:** fatty acid amide hydrolase (FAAH), N-acylethanolamine acid amidase (NAAA), carmofur, acute lung injury, drug repurposing

## Abstract

Acute lung injury (ALI), characterized by a severe inflammatory process, is a complex syndrome that can lead to multisystem organ failure. Fatty acid amide hydrolase (FAAH) and *N*-acylethanolamine acid amidase (NAAA) are two potential therapeutic targets for inflammation-related diseases. Herein, we identified carmofur, a 5-fluorouracil-releasing drug and clinically used as a chemotherapeutic agent, as a dual FAAH and NAAA inhibitor. In Raw264.7 macrophages, carmofur effectively reduced the mRNA expression of pro-inflammatory factors, including IL-1β, IL-6, iNOS, and TNF-α, and down-regulated signaling proteins of the nuclear transcription factor κB (NF-κB) pathway. Furthermore, carmofur significantly ameliorated the inflammatory responses and promoted resolution of pulmonary injury in lipopolysaccharide (LPS)-induced ALI mice. The pharmacological effects of carmofur were partially blocked by peroxisome proliferator-activated receptor-α (PPARα) antagonist MK886 and cannabinoid receptor 2 (CB2) antagonist SR144528, indicating that carmofur attenuated LPS-induced ALI in a PPARα- and CB2-dependent mechanism. Our study suggested that carmofur might be a novel therapeutic agent for ALI, and drug repurposing may provide us effective therapeutic strategies for ALI.

## Introduction

Acute lung injury (ALI) or its more severe form acute respiratory distress syndrome (ARDS) is a spectrum of lung diseases characterized by a severe inflammatory process, resulting in severe hypoxemia, hypercapnia, diffuse infiltration, and poor pulmonary compliance. ALI may lead to multisystem organ failure and is still a life-threatening disease in critically ill patients (Rubenfeld et al., 2005). Patients diagnosed with ALI are often treated with mechanical ventilation during the course of illness. Although mechanical ventilation is helpful for resolving life-threatening hypoxia and hypercapnia, clinical and experimental studies have shown that mechanical ventilation, if performed incautiously, will further damage the lungs due to overinflation, varotrauma, as well as cyclic closing and reopening of the alveoli, causing ventilator-associated lung injury (Diaz et al., 2010). Moreover, the mechanism of ventilator-associated lung injury may trigger a pulmonary and systemic inflammatory reaction that may further lead to multiple organ dysfunction or multiple system organ failure. Over the past decades, despite considerable progress has been made in understanding the pathogenesis and pathophysiology of ALI, there is still no effective therapeutic strategy for these diseases.

Trauma, pneumonia, acid aspiration, and sepsis resulting from Gram-negative pathogenic bacteria infection are the most common causes of ALI. During acute pulmonary infection, the Toll-like receptor-4 (TLR-4) on epithelial cells and alveolar macrophages detect lipopolysaccharide (LPS), a major component of the outer cell membrane of bacteria, and elicit innate immune responses that eliminate the invading microorganisms (Hussell and Bell, 2014; Kumari et al., 2015). TLR-4 stimulates epithelial and immune cells to produce chemokines and pro-inflammatory cytokines by activation of the nuclear transcription factor κB (NF-κB) pathway, and recruit neutrophils into the lung (Wheeler and Bernard, 1999; Miyake, 2004). Although the engulfed neutrophils kill bacteria during the immune response, it also produces large amounts of free radicals and reactive oxygen species. Uncontrolled inflammation and excessive accumulation of neutrophils can cause alveolar barrier damage and lung dysfunction, leading to respiratory failure and death (Wheeler and Bernard, 1999; Santus et al., 2018). Therefore, regulation of inflammation is critical in protecting ALI, and anti-inflammatory treatment has been proposed as a promising therapeutic strategy for ALI.

Anandamide (AEA), oleoylethanolamide (OEA), and palmitoylethanolamide (PEA) are classic endogenous fatty acid ethanolamides (FAEs) exhibiting anti-inflammatory and analgesic activities. AEA suppresses the inflammatory process by activating cannabinoid receptor 1 (CB1) and CB2, while OEA and PEA exert anti-inflammatory effects through interaction with the nuclear peroxisome proliferator-activated receptor-α (PPARα) (Lo Verme et al., 2005; Pandey et al., 2009; Petrosino et al., 2010; Yang et al., 2016). Fatty acid amide hydrolase (FAAH) and *N*-acylethanolamine acid amidase (NAAA) are the important enzymes in the degradation of FAEs. Pharmacological blocking of NAAA or FAAH increases endogenous FAE levels in rodent models, exhibiting analgesic and anti-inflammatory effects (Ahn et al., 2009; Bottemanne et al., 2018; Zhou et al., 2019). Inhibition of FAAH and NAAA showed no undesirable cardiovascular effects and gastrointestinal hemorrhaging as commonly seen with cyclo-oxygenase-2 (COX-2) inhibitors (Yang et al., 2015; Ren et al., 2017), and have become an alternative therapeutic strategy for inflammation-related diseases.

Both FAAH and NAAA inhibitors have shown beneficial actions in pulmonary diseases and are potential therapeutic strategies for ALI. FAAH inhibitor URB937 can attenuate one-lung ventilation (OLV)-induced lung injury, with an increase in the arterial oxygenation index and decreases in the lung injury score (Yin et al., 2019). NAAA inhibitor ARN726 and F215 also display pronounced therapeutic effects and reduce the tissue damages by suppressing inflammation during the lung injury (Ribeiro et al., 2015; Li et al., 2018). However, it is extremely challenging to push these compounds towards clinical application. One approach to expediting drug development is to discover novel applications for clinically approved drugs, i.e., drug repurposing. With well-known therapeutic window and adverse effects, the old drugs can rapid enter phase II clinical trials. Therefore, we now direct our attention towards clinical drugs and focus on identifying potent anti-inflammatory agents from old drugs.

Carmofur, an antineoplastic drug that has been clinically used for more than 37 years, is also known as an inhibitor of acid ceramidase (AC) (Taguchi, 1980; Kikuchi and Uekama, 1988; Furukawa et al., 2000). AC, a lysosomal enzyme mainly responsible for ceramide degradation, plays an important role in the control of cancer cell proliferation (Realini et al., 2016). We speculated that AC inhibitor carmofur may suppress NAAA activity, since AC exhibits 33–35% amino acid identity with NAAA. Additionally, carmofur contains a urea group, which is commonly seen in many potent FAAH and NAAA inhibitors (Tuo et al., 2017). Carmofur is likely to act as an FAAH and NAAA inhibitor, exhibiting anti-inflammatory activities by regulating the levels of FAEs. As a proof of concept, we examined the capability of carmofur to inhibit FAAH and NAAA, and its therapeutic effects in the LPS-induced lung injury. Our results show that carmofur is an FAAH and NAAA dual inhibitor, and administration of carmofur exhibited beneficial actions in lung injury through a PPARα- and CB2-dependent pathway.

## Materials and methods

### Chemicals

All reagents used in the present study were purchased from Sigma (Shanghai, China), seeking the highest grade commercially available unless otherwise indicated. Carmofur, MK886, and SR144528 were purchased from Cayman Chemical (Michigan, USA).

### *In Vitro* Biological Evaluation of Carmofur

#### Cell Culture

HEK293 cells overexpressing rat NAAA (HEK293-rNAAA) and rat FAAH (HEK293-rFAAH) and mouse macrophage Raw264.7 cells were plated and cultured in DMEM supplemented with 10% FBS and 2 mM glutamine overnight until 80% confluence. Raw264.7 cells were then incubated with carmofur of a series of concentrations for 30 min followed by challenging with LPS (*Escherichia coli* 0111:B4, Sigma, 500 ng/ml) for 72 h (Lee et al., 2008; Li et al., 2012).

#### Immunofluorescence Staining for p65

Raw264.7 cells were cultured on coverslips under the same conditions as described above. The cells were then fixed with acetone at −20°C for 30 min and subsequently permeabilized with 0.2% Triton X-100 for 5 min at room temperature. The cells were then incubated overnight at 4°C with primary antibody (Anti-p65, Abcam, ab32536, dilution 1:400) (Du et al., 2017), rinsed with 0.1 M phosphate-buffered saline (PBS), and exposed to goat anti-rabbit IgG 647 (Abcam, ab150079, dilution 1:1,000) for 2 h (Du et al., 2017). After washing in PBS, the cells were incubated with 4′,6-diamidino-2-phenylindole (DAPI) for 5 min, mounted by VECTASHIELD mounting medium, and then observed under a confocal microscope (Olympus, Japan). The translocation rate was counted from all fields (Stojak et al., 2018; Yao et al., 2018).

#### Real-Time Quantitative PCR

Total RNA was extracted from Raw264.7 cells with TRIzol (Invitrogen) and quantified by spectrophotometer (Beckman Coulter, Shanghai, China). cDNA was synthesized from 1 μg of total RNA by using the ReverTra Ace qPCR RT Kit (TOYOBO, Shanghai, China) following the manufacturer’s instructions. Real-time quantitative PCR was performed in a 7300 real-time PCR System (Applied Biosystems, CA, USA) using SYBR Premix Ex Taq GC (Takara, Dalian, China). The amplification steps included denaturation at 95°C for 30 s (1 cycle), annealing at 60°C for 60 s (1 cycle), and extension at 72°C for 60 s (26 cycles). RNA levels were normalized using glyceraldehyde-3-phosphate dehydrogenase (GAPDH) as a reference gene.

The primer sequences for mouse genes were as follows:

IL-6: 5′-ACAAGTCGGAGGCTTAATTACACAT-3′ (forward), 5′-TTGCCATTGCACAACTCTTTTC-3′ (reverse);
IL-1β: 5′-TCGCTCAGGGTCACAAGAAA-3′ (forward), 5′-CATCAGAGGCAAGGAGGAAAAC-3′ (reverse);
TNF-α: 5′-AGCCCCCAGTCTGTATCCTT-3′ (forward), 5′-GGTCACTGTCCCAGCATCTT-3′ (reverse);
iNOS: 5′-GGCAGCCTGTGAGACCTTTG-3′ (forward), 5′-GCATTGGAAGTGAAGCGTTTC-3′ (reverse);
GAPDH: 5′-GGTGAAGGTCGGTGTGAACG-3′ (forward), 5′-CTCGCTCCTGGAAGATGGTG-3′ (reverse).

#### Protein Preparation and Enzymatic Assay

HEK293-rNAAA and HEK293-rFAAH cells were incubated with carmofur of a series of concentrations for 30 min. The cells were harvested, washed with PBS, sonicated in 20 mM Tris–HCl (pH 7.5) containing 0.32 M sucrose, and then centrifuged at 800 × *g* for 15 min at 4°C. The supernatants were collected and subjected to three freeze–thaw cycles at −80°C, and protein concentrations were measured by the BCA protein assay kit. The crude protein containing FAAH or NAAA was used as such without further purification.

FAAH activity was measured by incubating protein (30 μg) with AEA (25 mM) in 0.2 ml Tris–HCl buffer (50 mM, pH 8.0, containing 0.05% fatty acid-free BSA) at 37°C for 30 min. NAAA activity was measured by incubating protein (30 μg) with heptadecenoylethanolamide (C17:1 FAE) (25 mM) in 0.2 ml phosphate buffer (50 mM, pH 5.0, containing 0.1% Triton X-100, and 3 mM DTT) at 37°C for 30 min. The reactions were terminated by adding methanol (0.2 ml, containing 1 nmol heptadecanoic acid as internal standards), and the residual hydrolysis products of the substrate were determined by HPLC-MS. NAAA inhibitor (S)-OOPP and FAAH inhibitor URB597 were used as the positive control.

#### Lipid Extraction and Analysis

Lipids were extracted from Raw264.7 cells and tissue samples using a previously described method (Li et al., 2017). Samples were homogenized in CH_3_OH-H_2_O (1:1) containing C17:1 FAE as internal standards and then extracted with CHCl_3_. The organic phase was dried under N_2_ flow, and the residue was purified by solid-phase extraction using CH_3_OH-CHCl_3_ (1:9) as eluent. The eluate was dried and reconstituted in methanol (100 μl), and the CH_3_OH solution (10 μl) was used for LC-MS/MS assay. The mobile phase consisted of CH_3_OH and H_2_O (pH 7.4), and the gradient elution was as follows: 85% CH_3_OH for the first 3 min, followed by a linear gradient from 85% to 100% CH_3_OH for 2 min, and then 100% CH_3_OH for 15 min. The elution condition was finally returned to 85% CH_3_OH at a flow rate of 0.7 ml/min. The column temperature was maintained at 40°C. Ion detection was monitored by APCI^+^-MRM mode. The molecular ions were measured at *m*/*z* 348.00/62.00 for AEA, *m*/*z* 300.3/62.0 for PEA, and *m*/*z* 313.1/62.0 for C17:1 FAE.

#### Western Blot

Total protein (50 μg) was loaded onto a 10% sodium dodecyl sulfate (SDS)–polyacrylamide gel, electrophoresed for 2 h at 100 V, and then transferred to Hybond-P membranes (Amersham Biosciences, Shanghai, China). Membranes were blocked in 3% skimmed milk for 1 h at room temperature and then incubated with antibodies against p-p65 (Abcam, ab32536, dilution 1:1,000) or p-IκBα (Abcam, ab32518, dilution 1:2,000) (Du et al., 2017; Vander Lugt et al., 2017). Bands were visualized with an electrochemiluminescence plus kit (Amersham Biosciences). Quantitative analyses were performed using Image J software, with α-tubulin as the internal standard.

#### Competition Binding Assay

The binding affinity of carmofur to PPARα was detected by PPARα LanthaScreen™ TR-FRET competitive binding assay (Invitrogen) following the kit instruction. Carmofur (20 μM), GW7647 (positive control, 10 μM), or DMSO (negative control) was incubated with a mixture of the GST-tagged PPARα ligand-binding domain (LBD), terbium-labeled anti-GST antibody, and a green fluorescent pan-PPAR ligand (tracer). The mixture was gently shaken for 30 s and then incubated at 25°C for 5 h. The fluorescent emission intensity at 520 nm (λex = 495 nm) was measured.

The affinities of carmofur for CB2 receptor was determined by a competitive radioligand displacement assay. Carmofur (20 μM), (R)-(+)-WIN 55212-2 (positive control, 5 μM) or DMSO (negative control) was incubated with [^3^H]-CP55940 (1 nM) and the membrane protein (6 µg) from CB2 overexpressing Chinese hamster ovary (CHO) cells in binding buffer (50 mM Tris–HCl, 5 mM MgCl_2_, 2.5 mM EDTA, and 0.5 mg/ml BSA, pH 7.4). The mixture was incubated at 25°C for 2 h, rapidly filtered over a UniFilter-96 GF/C glass fiber plate, and washed with ice-cold Tris–HCl (50 mM, pH 7.4). The radioactivity on the filters was measured with the TopCount NXT microplate scintillation counter (PerkinElmer) using 30 µL of MicroScint 40 (PerkinElmer).

### *In Vivo* Biological Evaluation of Carmofur

All animal experiments were performed in accordance with the Guide for the Care and Use of Laboratory Animals from the National Institutes of Health (NIH) and approved by the Animal Care and Use Committees of Xiamen University in China.

#### LPS-Induced ALI

The LPS-induced ALI model was used to test the anti-inflammatory activity of carmofur (Li et al., 2018). Male C57BL/6J mice (20–22 g) were randomly grouped, with eight animals for each group. Mice were anesthetized with chloral hydrate and instilled intratracheally with LPS (5 mg/kg). Carmofur [3 and 10 mg/kg, dissolved in saline with 5% polyethylene glycol 400 (PEG400) and 5% Tween 80] or its vehicle was orally administered twice a day starting from the day of LPS application. Mice were sacrificed 0, 1, and 3 days after LPS instillation.

#### Analysis of Bronchoalveolar Lavage Fluid (BALF)

On days 0, 1, and 3, mice were euthanized, and lungs were infused five times with 1 ml of PBS (pH 7.5). BALF was extracted, combined, and then centrifuged at 1500 rpm for 10 min at 4°C. The protein content in the supernatant was measured using a bicinchoninic acid (BCA) kit (Pierce). The amount of IL-1β and TNFα was measured in BALF using the murine IL-1β and TNFα ELISA kit according to the manufacturer’s instructions. The pellet was resuspended in PBS, and cell differentials were counted by using cytospin and Wright–Giemsa staining.

#### Evans Blue Dye Extravasation

Evans blue dye extravasation was measured following a previous reported method (Li et al., 2018). Mice were injected intravenously with Evans blue (40 mg/kg) and were euthanized 60 min later. Lungs were perfused to remove intravascular dye, followed by excision and homogenization in PBS. The lung homogenate was diluted with 2 volumes of formamide and incubated at 60°C for 18 h. After centrifugation at 2,000 × *g* for 30 min, the supernatant was collected and measured at wavelengths of 620 nm and 740 nm using a Tecan reader. The extravasated Evans blue dye concentrations were then calculated using the following formula: *A*_620_ (corrected) = *A*_620_ (actual) − (1.193 × *A*_740_) + 0.007.

#### Myeloperoxidase (MPO) Activity

MPO activity was measured using a previous reported method (Li et al., 2018). Lung samples were homogenized in a potassium phosphate buffer containing 0.5% hexadecyltrimethylammonium bromide, 0.5 mM EDTA, and then subjected to two freeze–thaw cycles at −20°C followed by centrifugation again at 12,000 × *g* for 30 min at 4°C. Supernatants were collected and added to MPO assay buffer containing potassium phosphate buffer (50 mM, pH 6), H_2_O_2_ (w/v, 0.0005%) and o-dianisidine dihydrochloride (0.168 mg/ml). After 25 min of incubation at 25°C, the samples were read at 460 nm for 3 min. The absorbance change during the 30- to 90-s time course was recorded. MPO activity was then calculated using the following formula: MPO activity (U/g) = Δ*A*_460_ × 13.5/lung wet weight (g).

#### Histology

The whole lungs of mice were harvested and fixed in paraformaldehyde at 4°C, followed by embedding in paraffin. Sections with a thickness of 5 μm were obtained by a microtome. The slices were then stained with hematoxylin and eosin (H&E) after deparaffinized with xylene. Imaging of stained areas was performed with a light microscope (Nikon, Shanghai, China) using ×4 objectives (Dong et al., 2018).

The H&E-stained image was analyzed in a semiquantitative fashion following a literature procedure with minor modification (Zeldin et al., 2001): The evaluations were recorded as the perivascular edema (P1), the perivascular acute inflammation (P2), goblet-cell metaplasia of bronchioles (P3), and eosinophilic macrophages in alveolar spaces (P4). P1 was assessed using the following criteria: 0 = no change; 1 = < 25% of the perivascular spaces; 2 = 25%–75% of the perivascular spaces; 3 = > 75% of the perivascular spaces. P2 was assessed using the grading criteria of inflammatory cells surrounded veins: 0 = absent; 1 = few; 2 = a thin layer (1–5 cells thick); 3 = a thick layer (>5 cells thick). P3 was assessed using the grading criteria of goblet cells present in one or two bronchiolar profiles: 0 = absent; 1 = few; 2 = large. P4 was assessed using the grading criteria of eosinophilic macrophages in alveolar spaces: 0 = absent; 1 = < 25%; 2 = > 25%. The total inflammatory score (range 0–10) taken as the sum of the individual scores, were then calculated using the following formula: *H* score = P1 + P2 + P3 + P4. All of the histology samples were scored blindly and independently by at least two investigators.

#### Immunohistochemistry of Lungs Samples

Immunohistochemical analysis was performed on paraffin-embedded lung samples. Heat-induced epitope retrieval (Haier, microwave treatment, 600 W) was performed using prewarmed sodium citrate buffer (10 mM, pH 6.0) for 2 × 15 min. In order to block endogenous peroxidase activity, samples were treated with 3% H_2_O_2_/CH_3_OH for 10 min, permeabilized with 0.1% Triton X-100 in PBS for 20 min, and then washed in PBS. Next, slides were blocked with goat serum for 30 min to prevent non-specific binding, followed by incubation overnight at 4°C in the presence of primary antibodies (IL-1β, Abcam, ab200478, dilution 1:100; IL-6, Cell Signaling Technology, 5216, dilution 1:100; TNF-α, Abcam, ab6671, dilution 1:100) (Ji et al., 2018). Sections were then washed with 0.1 M PBS and incubated in the presence of biotinylated goat anti-rabbit IgG secondary antibody (Vector Lab, dilution 1:400) for 1 h at 37°C, followed by incubation with a Vectastain Elite ABC reagent (Vector Lab, dilution 1:200) for 1 h, and then incubated in 2% nickel intensified diaminobenzidine (DAB) for 10 min. Images were taken with an Olympus microscope at 10× magnification. The number of positively stained cells in the whole cartilage area per specimen in five sequential sections was quantified by Image Pro Plus (Media Cybernetics). To confirm the antibody binding specificity for IL-6, IL-1β, and TNF-α, some sections were also incubated with primary or secondary antibody only as controls.

The immunohistochemical image was analyzed in a semiquantitative fashion by a previously reported method with minor modification (McCarty et al., 1986). The evaluations were recorded as the overall stain intensity (P1) and the capability of carmofur in inhibiting (P2). The overall score of the staining intensity (P1) typically has four tiers ranging from 0 to 3: 0 = no staining; 1 = weak but detectable above control; 2 = distinct; 3 = strong; 4 = minimal light transmission through stained nucleus. The percentage of cells stained (P2) was assessed using the following criteria: 0 = 0%–5%; 1 = 6%–25%; 2 = 26%–50%; 3 = 51%–75%; 4 = > 75%. The immunohistochemical scores (*H* score) were then calculated using the following formula: *H* score = P1 + P2. All of the histology samples were scored blindly and independently by at least two investigators.

#### Pharmacokinetics Study

Carmofur were administered orally to C57BL/6J mice with doses of 3 and 10 mg/kg. Three animals per dose and per time point were used. Mice were sacrificed and lung samples were collected at 0, 5, 15, 30, 45, 60, 120, 240, 480, and 720 min after drug administration. Lung tissues (20–30 mg) were homogenized by ultrasonication in 0.5% HCl solution (1 ml), followed by extraction with chloroform (3 × 3 ml) and then vortex for 1 min. Chloroform layer was separated by centrifugation at 3,000 × g for 10 min and then transferred to a clean V-bottom glass tube and dried under nitrogen (N_2_) flow. Chloroform (1 ml) was added to resolve dried spots and solid-phase extraction was eluted by methanol/chloroform (v/v, 1/9). The elution containing carmofur was dried under N_2_ and reconstituted in CH_3_CN for HPLC analysis. Briefly, 20 μl of each sample was injected into a reversed-phase column (Agilent, C18 4.6 × 250 mm, 5 μm particle size). Carmofur was eluted by CH_3_CN/H_2_O (50/50, v/v, 0.25% phosphoric acid) with a flow rate of 1.0 ml min^−1^ at 40°C and determined by UV detection at 260 nm. Carmofur in the lung homogenate with concentrations ranging from 500 to 20,000 ng/ml was prepared for the external standard curve (Kobari et al., 1978).

### Data and Statistical Analysis

Randomization was used to assign mice to different experimental groups, to collect and process data. All histology samples were scored blindly and independently by at least two investigators. All statistical analyses were completed using GraphPad Prism version 5.01. Data are shown as the mean ± SEM. Three or more different groups were analyzed by one-way ANOVA with Dunnett’s *post hoc* multiple comparison tests. For all ANOVA, *post hoc* tests were run only if *F* achieved *P* < 0.05 and there was no significant variance inhomogeneity. *P* < 0.05 was considered statistically significant.

## Results

### Carmofur is an FAAH and NAAA Dual Inhibitor

**Figure 1:**
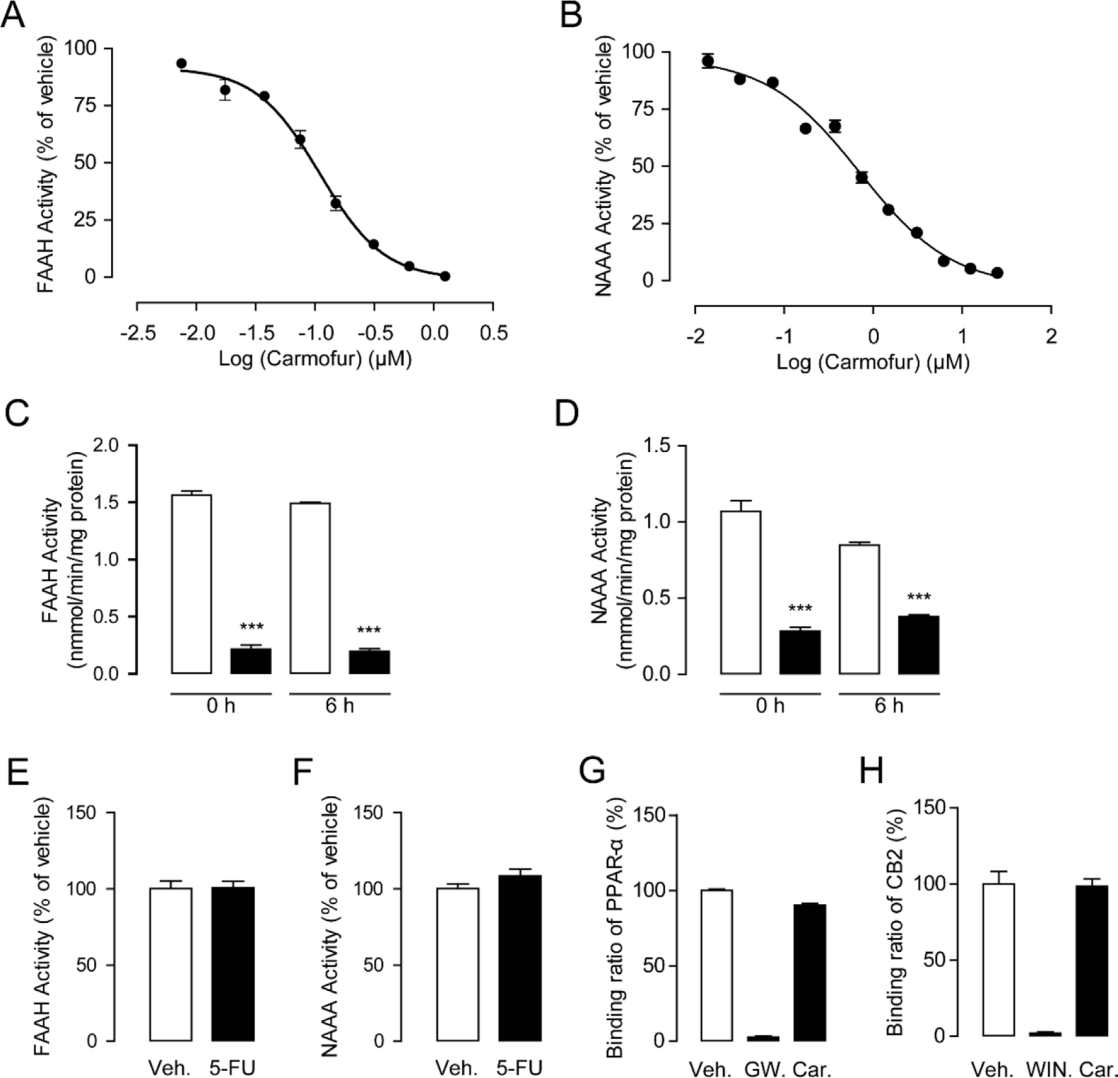
Characterization of carmofur as a fatty acid amide hydrolase (FAAH) and N-acylethanolamine acid amidase (NAAA) dual inhibitor. Concentration-dependent inhibition of HEK293-rFAAH **(A)** and HEK293-rNAAA **(B)** by carmofur. Effects of dialysis (0, 6 h; 0–4°C) on the activities of FAAH **(C)** and NAAA **(D)** by carmofur (5 µM). Results are expressed as mean ± SEM, *n* = 3 replicates per group. ***P < 0.001 vs. vehicle control by one-way ANOVA. Effect of vehicle (1% DMSO) or 5-fluorouracil (20 µM) on FAAH **(E)** and NAAA **(F)** activities in HEK293 cells heterogeneously overexpressing FAAH and NAAA. Competition binding assay of vehicle (1% DMSO), GW7647 (10 µM), (R)-(+)-WIN 55212-2 (5 µM), and carmofur (20 µM) for PPARα **(G)** and CB2 **(H)**.

As shown in **Figures 1A, B**, carmofur exhibited potent inhibitory activities towards rat recombinant FAAH (IC_50_ = 0.11 ± 0.025 μM) and NAAA (IC_50_ = 0.71 ± 0.054 μM) in FAAH or NAAA overexpressed HEK293 cells. However, 5-fluorouracil (5-FU), the active form of carmofur in chemotherapy, showed no inhibitions towards FAAH and NAAA (**Figures 1E, F**). The hydrolase activities of the carmofur–rFAAH and carmofur–rNAAA interaction complexes were not recovered 6 h after dialysis, suggesting that carmofur may inhibit FAAH and NAAA in an irreversible pattern (**Figures 1C, D**). To determine whether carmofur can directly regulate the activities of CB2 and PPARα receptors, we examined its ability to bind to CB2 and PPARα receptors by the receptors’ competitive binding assay. The results suggested that carmofur did not bind to the ligand-binding domain (LBD) of PPARα and CB2 even at a high dose of 20 μM (**Figures 1G, H**). Taken together, these results suggested that carmofur was a potent and irreversible FAAH and NAAA dual inhibitor, and it cannot directly active PPARα and CB2.

### Effect of Carmofur on LPS-Induced Macrophage Activation

**Figure 2:**
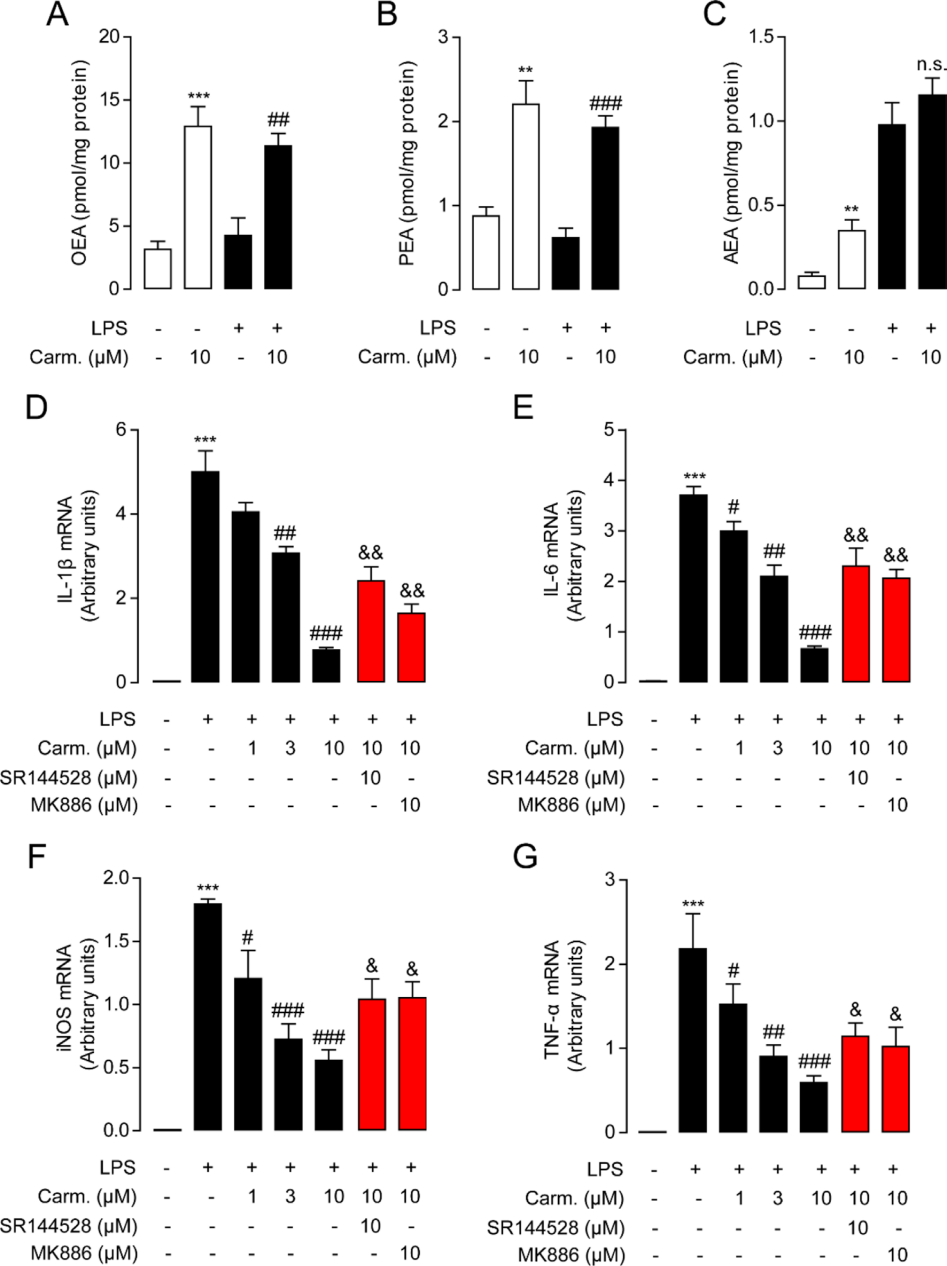
Carmofur reduced lipopolysaccharide (LPS)-induced inflammation in Raw264.7 cells. Effect of carmofur or vehicle on palmitoylethanolamide (PEA) **(A)**, oleoylethanolamide (OEA) **(B)**, and anandamide (AEA) levels **(C)** in Raw264.7 cells. Effect of carmofur, SR144528, and MK886 on mRNA expression of IL-1β **(D)**, IL-6 **(E)**, iNOS **(F)**, and TNF-α **(G)** in Raw264.7 cells treated with vehicle (0.1% DMSO) or LPS (500 ng/ml) for 72 h. Results are expressed as mean ± SEM, *n* = 3 replicates per group. mRNA levels are presented relative to the GAPDH mRNA expression. **P < 0.01; ***P < 0.001 vs. Sham. ^#^ *P* < 0.05; ^##^ *P* < 0.01; ^###^ *P* < 0.001 vs. LPS + vehicle. ^&^ *P* < 0.05; ^&&^ *P* < 0.01 vs. LPS + carmofur (10 µM) by one-way ANOVA test.

As macrophages take up most of the LPS in the lungs during pulmonary infection (Coers et al., 2017), we next investigated the influence of carmofur on the levels of FAEs in Raw264.7 cells. Carmofur (10 μM) alone significantly suppressed FAAH and NAAA activities and increased the levels of all three FAEs in Raw 264.7 cells (**Figures 2A–C**, **Figure S1**). However, in LPS-treated Raw 264.7 cells, carmofur failed to further elevate the AEA levels (**Figure 2C**). We then examined whether carmofur modulated the LPS-induced inflammatory responses in Raw264.7 cells. Real-time quantitative PCR and ELISA analysis demonstrated that LPS treatment increased expression of inflammatory cytokines, including IL-1β, IL-6, iNOS, and TNF-α. The increase in cytokine mRNA and protein expression was dose-dependently suppressed by carmofur (1–10 μM) (**Figures 2D–G** and **S2**). Moreover, the anti-inflammatory effect of carmofur on Raw264.7 cells was blocked by PPARα antagonist MK886 (10 μM) or CB2 antagonist SR144528 (10 μM), indicating that PPARα and CB2 pathways are involved in the anti-inflammatory action of carmofur in macrophages (**Figures 2D–G** and **S2**).

**Figure 3:**
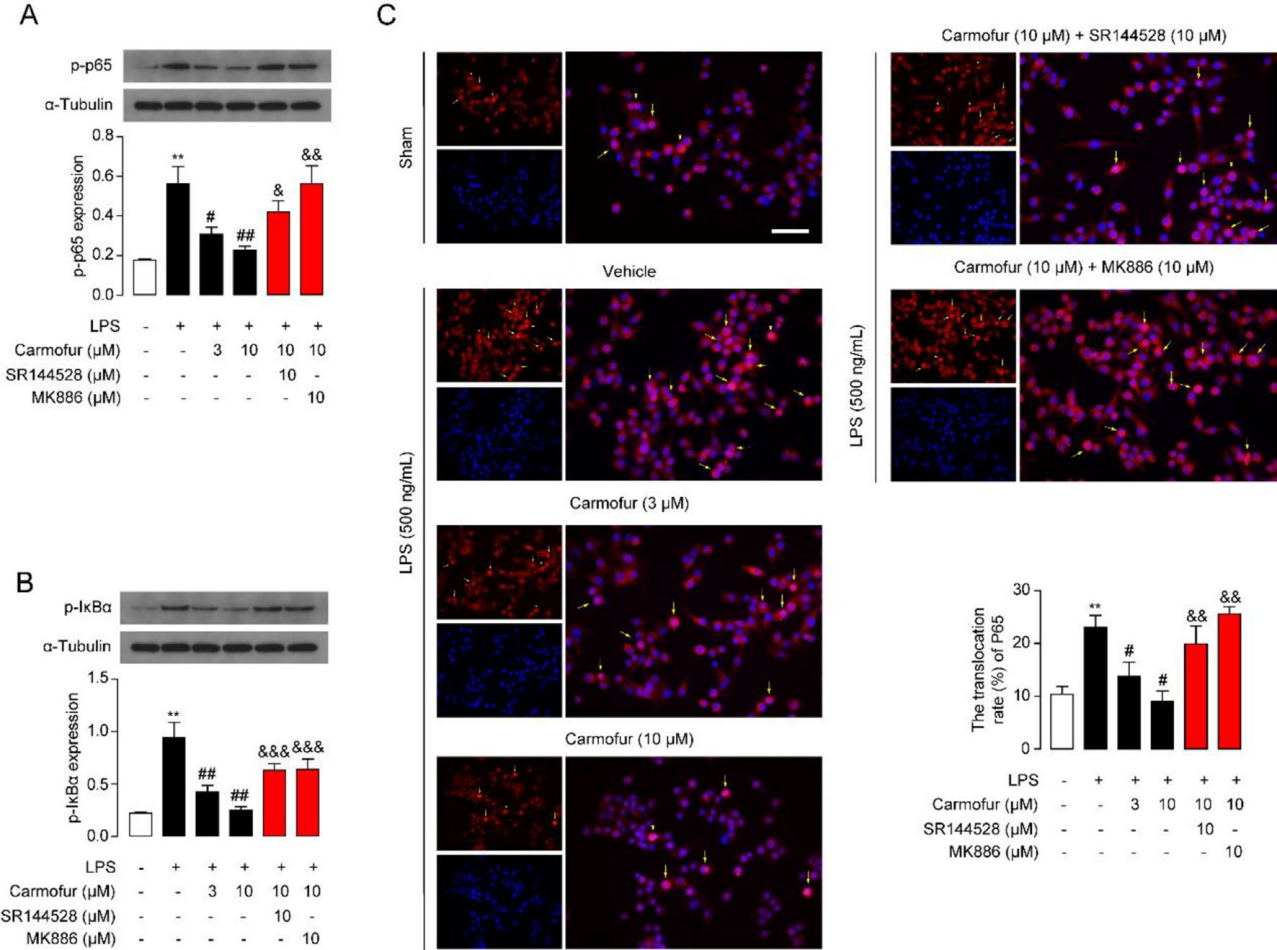
Carmofur suppressed nuclear transcription factor κB (NF-κB) signaling in Raw264.7 cells. Representative Western blots band and quantification of p-p65 **(A)** and p-IκBα **(B)** abundances in Raw264.7 cells treated with vehicle, carmofur, SR144528 or MK886 followed by challenging with vehicle (0.1% DMSO) or LPS (500 ng/ml) for 72 h. Results are expressed as mean ± SEM, *n* = 3 replicates per group. **P < 0.01 vs. sham. ^#^ *P* < 0.05; ^##^ *P* < 0.01 vs. LPS + vehicle. ^&^ *P* < 0.05; ^&&^ *P* < 0.01; ^&&&^ *P* < 0.001 vs. LPS + carmofur (10 µM) by one-way ANOVA test. **(C)** Representative confocal imaging of p65 in Raw264.7. Red, p65; blue, nucleus; Yellow arrows indicate nuclear translocation of p65. Scale bars, 50 µm.

The NF-κB pathway is an important signaling pathway involved in inflammation response in ALI; therefore, we further explored whether carmofur affected the cascade of signaling proteins related to the NF-κB pathway. As shown in **Figure 3**, LPS stimulation increased the phosphorylation of NF-κB p65 (p-p65) and IκBα (p-IκBα), and enhanced the nuclear translocation of p65 in Raw264.7 cells. Carmofur dose-dependently down-regulated the protein expression levels of p-p65 and p-IκBα, and blocked the nuclear translocation of p65 (3 and 10 μM) (**Figures 3A–C**). Additionally, the PPARα antagonist MK886 (10 μM) and CB2 antagonist SR144528 (10 μM) both partly abolished the anti-inflammatory effects of carmofur in Raw264.7 cells (**Figures 3A–C**). These results suggested that carmofur may alleviate LPS-induced inflammation through the NF-κB signaling pathway, probably mediated by PPARα and CB2 receptors.

### Biological Stability of Carmofur in Mice

**Figure 4:**
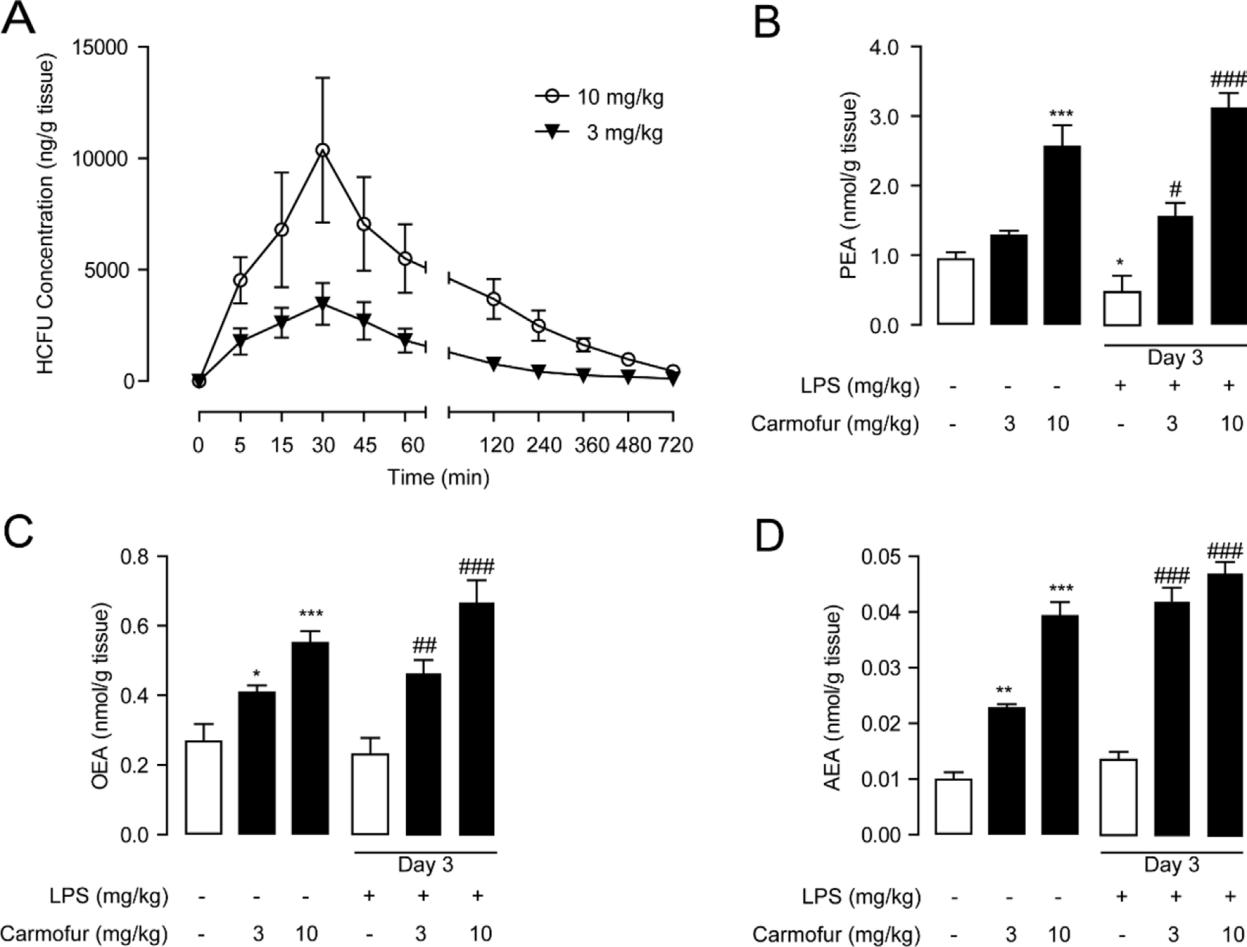
Pharmacokinetic and pharmacodynamic studies of carmofur in mice. **(A)** Levels of carmofur in lungs at the different time points after oral administration (3 and 10 mg/kg). Levels of PEA **(B)**, OEA **(C)**, and AEA **(D)** in lungs in mice 2 h after oral administration of carmofur (3 and 10 mg/kg) or its vehicle (5% PEG/5% Tween-80 in saline). Results are expressed as mean ± SEM (*n* = 8 mice per group). *P < 0.05; **P < 0.01; ***P < 0.001 vs. sham group; ^#^ *P* < 0.05; ^##^ *P* < 0.01; ^###^ *P* < 0.001 vs. LPS + vehicle group by one-way ANOVA test.

In previous studies, carmofur showed poor stability in mouse plasma *in vitro* (Pizzirani et al., 2013); thus, we further examined its stability in the lung. Carmofur were administered orally to C57BL/6J mice, and the drug concentrations in the lung at different time points were determined by HPLC. As shown in **Figure 4A**, carmofur reached peak levels of 3,460 ng/g tissue (∼13 μM) and 10,368 ng/g tissue (∼40 μM) in the lung at 30 min after administration at doses of 3 and 10 mg/kg, respectively, and showed a half-life (*T*_1/2_) of nearly 45 min in the lung. Interestingly, 8 h after drug administration, considerable amount of carmofur was detected in the lung with concentrations of 190 ng/g tissue (∼0.7 μM) and 980 ng/g tissue (∼3.8 μM) at doses of 3 and 10 mg/kg, respectively. It is possible that carmofur bound to proteins in lungs and thus improved the compound biological half-life. Furthermore, oral administration of carmofur dose-dependently increased PEA, OEA, and AEA levels in lungs (**Figures 4B–D**). Taken together, these data indicated that carmofur was relatively stable in the lung and was suitable for systemic administration.

### Effect of Carmofur on LPS-Induced ALI in Mice

**Figure 5:**
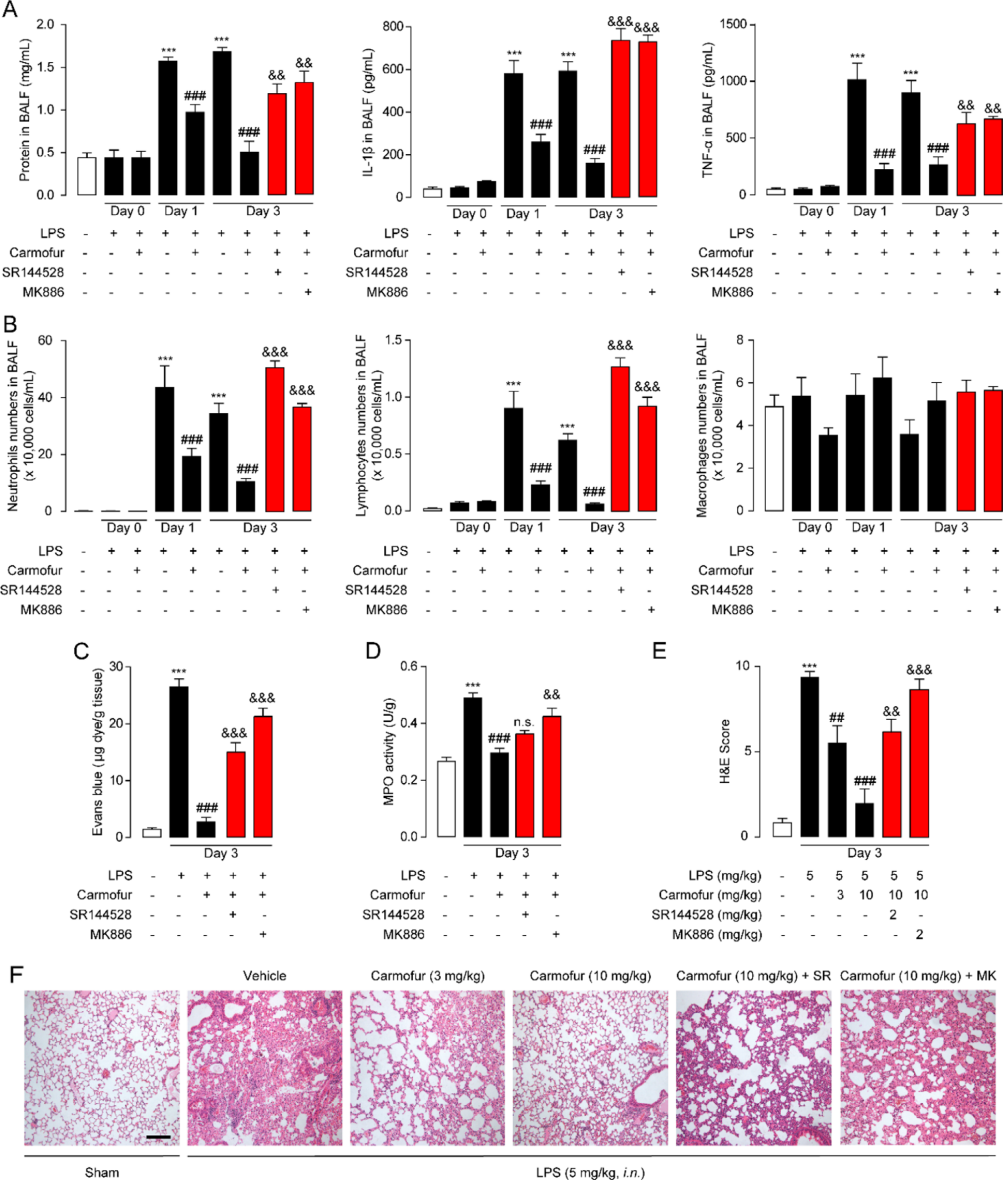
Carmofur ameliorated LPS-induced lung injury and promotes lung recovery. **(A)** Total protein, IL-1β, and TNF-α concentrations in BALF in ALI mice. **(B)** Immune cells in BALF were counted and differentially analyzed by using cytospin and Wright–Giemsa staining. Evans blue extravasations **(C)** and myeloperoxidase (MPO) activities **(D)** in lungs of LPS-induced ALI mice treated with carmofur (10 mg/kg, orally) or their vehicle. **(E** and **F)** Representative H&E-stained lung sections of sham mice, and vehicle-, carmofur (3 and 10 mg/kg, orally)-, SR144528 (2 mg/kg, i.p.)-, and MK886 (2 mg/kg, i.p.)-treated ALI mice sacrificed 3 days after LPS (5 mg/kg, i.n.). Results are expressed as mean ± SEM (*n* = 8 mice per group). **P < 0.01; ***P < 0.001 vs. sham. ^##^ *P* < 0.01; ^###^ *P* < 0.001 vs. LPS + vehicle. ^&&^ *P* < 0.01; ^&&&^ *P* < 0.001 vs. LPS + carmofur (10 µM) by one-way ANOVA test.

Encouraged by the *in vitro* anti-inflammatory activity and stability of carmofur in the lung, we then investigated whether carmofur could attenuate LPS-induced ALI. As shown in **Figures 4B–D**, LPS induced PEA decrease in lungs but had no effect on AEA and OEA levels. Carmofur (10 mg/kg, orally) effectively suppressed FAAH and NAAA activities (**Figure S1**) and increased the levels of all three FAEs in lungs in ALI mice, which was in agreement with the in vitro results. Accumulation of protein-rich fluid in alveolar spaces is an important symptom of pulmonary injury; thus, BALFs were collected and the protein concentrations were determined. As shown in **Figure 5A**, 1 day after LPS instillation, significantly elevated alveolar protein and inflammatory cytokine levels in BALF were observed for both vehicle-treated and carmofur-treated ALI mice, indicating the extravascular protein accumulation in the alveolar space. On day 3, the protein and inflammatory cytokine concentrations in BALF from carmofur-treated ALI mice returned to normal levels, while protein and cytokine abundances of BALF from the vehicle-treated group were still high. Immune cell recruitment to the alveolar spaces is another important symptom of lung inflammation. We also observed increased alveolar neutrophil and lymphocytes in BALF from ALI mice on days 1 and 3, while carmofur-treated ALI mice showed fewer immune cells compared to the vehicle-treated group (**Figure 5B**). However, there is no significant difference in the number of macrophage between ALI mice and the sham group (**Figure 5B**). The capability of carmofur to preserve the integrity of alveolar was also evaluated by Evans blue extravasation (**Figure 5C**). The results showed that on day 3, LPS-treated mice showed a more severe leakage of Evans blue in the lung compared with sham mice. Carmofur markedly prevented the increase in Evans blue extravasation. Additionally, the antagonists MK886 and SR144528 significantly inhibited the effects of carmofur on cell recruitment, BALF protein and cytokines, dye extravasation, and MPO activity. Collectively, these data suggested that carmofur reduced the damages to the tissue by suppressing inflammation during the lung injury in LPS-induced ALI mice.

Prolonged neutrophil accumulation is another cardinal feature of ALI, and clearance of neutrophils is associated with inflammation resolution and predicts a good outcome. Therefore, we further examined the neutrophil accumulation by testing the activity of MPO, a marker for neutrophil abundance. The results showed that ALI mice lungs displayed sustained high levels of MPO activity, which was markedly suppressed by carmofur (10 mg/kg, orally) (**Figure 5D**). The healing effect of carmofur on ALI mice was further evaluated by histological analysis. Vehicle-treated ALI mice showed infiltration of neutrophils and alveolar edema on day 3 after LPS administration (**Figures 5E, F**). However, ALI mice treated by carmofur (3 and 10 mg/kg, orally) exhibited thinner alveolar walls and less alveolar edema, and inhibited neutrophil accumulations (**Figures 5E, F**). The inhibition of carmofur on neutrophil accumulations was partially blocked by the PPARα antagonist MK886 (2 mg/kg, i.p.) or CB2 antagonist SR144528 (2 mg/kg, i.p.) (**Figures 5E, F**). Additionally, to exclude the possibility that the effects of MK886 observed in the present study were due to inhibition of 5-lipoxygenase activating protein (FLAP), we also tested carmofur in PPAR-α knockout mice. As shown in **Figure S3A**, carmofur had no significant effect on neutrophil accumulation in lungs of PPAR-α knockout mice exposed to intranasal LPS. Moreover, the anti-inflammatory effects of carmofur were also not blocked by FLAP inhibitor Quiflapon sodium (**Figure S3B**). These data further confirmed that PPAR-α mediates the effects of carmofur in ALI mice.

**Figure 6:**
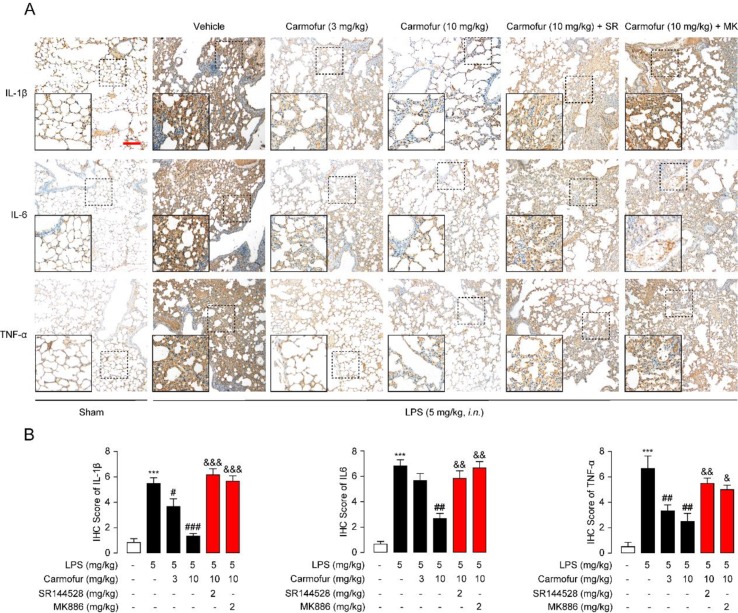
Carmofur reduced LPS-induced protein overexpression of inflammatory factors in lungs. **(A)** Representative immunohistochemical analyses of IL-1β, IL-6, and TNF-α in lung sections of sham mice, and ALI mice treated with vehicle (5% PEG/5% Tween-80 in saline, orally), carmofur (3 and 10 mg/kg, orally), SR144528 (2 mg/kg, i.p.), and MK886 (2 mg/kg, i.p.) sacrificed 3 days after LPS instillation. The images represent at least 75% of whole sections. Original magnification × 40; insets, × 100; scale bar, 250 µm. **(B)** Semiquantitative analysis of the immunohistochemical IL-1β, IL-6, and TNF-α expression. Results are expressed as mean ± SEM (*n* = 8 mice per group). ***P < 0.001 vs. sham. ^#^ *P* < 0.05; ^##^ *P* < 0.01; ^###^ *P* < 0.001 vs. LPS + vehicle. ^&^ *P* < 0.05; ^&&^ *P* < 0.01; ^&&&^ *P* < 0.001 vs. LPS + carmofur (10 µM) by one-way ANOVA test.

Finally, we studied the effect of carmofur on inflammatory cytokines *in vivo* by immunohistochemistry. LPS-treated ALI mice displayed persistently high levels of IL-1β, IL-6, and TNF-α in lungs, while carmofur but not vehicle dose-dependently reduced these up-regulated cytokines (**Figure 6**). Antagonist of CB2 or PPARα receptors blocked the anti-inflammatory effects of carmofur (**Figure 6**). Additionally, 5-FU (10 mg/kg, i.p.) had no significant effect on neutrophil accumulation and uncontrolled expression of inflammatory cytokines (data not shown). When combined, these data indicated that carmofur could reduce the damages to the tissue by suppressing inflammation during the lung injury in LPS-induced ALI mice.

## Discussion

Drug discovery and development is a lengthy and expensive process with high failure rate. It takes about $1–2 billion and 12–14 years to develop a new drug starting from the hit compound *in vitro*, and nearly 90% of drug candidates fail in clinical trials (Papapetropoulos and Szabo, 2018). Various studies suggested that FAAH and NAAA inhibitors are promising therapeutic agents for the treatment of inflammatory, pain, and other related diseases (Tuo et al., 2017; Bottemanne et al., 2018). In the past decade, many potent FAAH inhibitors have been developed, and some of them even have undergone clinical trials (Tuo et al., 2017). However, the results to date are rather disappointing. In these studies, PF04457845 developed by Pfizer failed to show therapeutic effects in humans, and a clinical trial of FAAH inhibitor (BIA 10-2474) was stopped because of its toxicity (Huggins et al., 2012; Report by the Temporary Specialist Scientific Committee (TSSC)). Most of the NAAA inhibitors described in literature are still under preclinical study until now (Bottemanne et al., 2018).

One way to expedite drug development is to discover new uses for approved or investigational drugs, i.e., drug repurposing. There are many successful examples for drug repurposing; e.g., gabapentin, originally being used as anti-epileptics, is now commonly used to treat neuropathic pain (Sharlow, 2016). Some clinically approved anti-inflammatory drugs have shown inhibitory activities towards FAAH (e.g., COX-2 inhibitors carprofen and ibuprofen) or NAAA (e.g., IL-1β antagonist diacerein) (Favia et al., 2012; Patel et al., 2013; Petrosino et al., 2015). Therefore, we switch our attention from newly synthesized FAAH and NAAA inhibitors to approved drugs to rapidly develop therapeutic agents for inflammation-related disease. As a proof of concept, carmofur, an antineoplastic drug, was selected, and the capability to attenuate ALI was evaluated in LPS-induced ALI mice. Carmofur is a known inhibitor of AC, an enzyme that exhibits 33–35% amino acid identity with NAAA; thus, we hypothesized that carmofur may also suppress NAAA activity. Our results demonstrated that carmofur is a potent FAAH and NAAA dual inhibitor. Carmofur effectively increased the levels of PEA, OEA, and AEA in macrophages in culture, reduced the mRNA expression of pro-inflammatory cytokines, and down-regulated signaling proteins of the NF-κB pathway. Moreover, carmofur reduced the tissue damages in LPS-induced ALI mice *via* ameliorating the inflammatory responses and suppressing neutrophils infiltration. The pharmacological effects of carmofur were blocked by CB2 antagonist SR144528 and PPARα antagonist MK886, indicating that carmofur attenuates ALI in a CB2- and PPARα-dependent pattern.

CB2 receptors, highly expressed in the periphery, mainly in immune cells (e.g., macrophages and mast cells), participate in the release of inflammatory cytokines involved in pulmonary injury (Liu et al., 2014). Previous reports showed that carrageenan and LPS-induced inflammation models exhibited CB2-dependent anti-inflammatory effects when FAAH was knocked out or blocked by inhibitors (Lichtman et al., 2004; Naidu et al., 2010; Booker et al., 2012). AEA is an agonist of CB2 receptor (Zygmunt et al., 1999; Di Marzo et al., 2004). Berdyshev et al. have shown that AEA could reduce neutrophil recruitment in the lung, at least in part, by CB2 receptor-mediated pathway in ALI mice (Berdyshev et al., 1998). Therefore, inhibition of the AEA catabolic enzymes FAAH and NAAA by carmofur significantly increased AEA levels in lungs and attenuated ALI. CB2 antagonist SR144528 partially blocked the pharmacological effects of carmofur, suggesting that CB2 may play an important role in the anti-inflammation effects *via* increasing AEA levels. In addition to increasing systemic AEA, carmofur also elevates levels of OEA and PEA, which are also mainly degraded by FAAH and NAAA. PEA and OEA exert anti-inflammatory *via* activating the PPARα receptor (Pontis et al., 2016). In agreement with a previous report that PEA effectively diminished the level of TNFα in BALF (Berdyshev et al., 1998), carmofur significantly reduced the up-regulated TNFα in ALI mice. The PPARα antagonist MK886 partly abolished the therapeutic effects of carmofur in ALI mice, suggesting that PPARα is also involved in the anti-inflammatory action of carmofur.

It is likely that the beneficial action of carmofur in ALI mice is from AC inhibition, since carmofur is a potent AC inhibitor. However, the available literature allows us to exclude this possibility. It has been reported that AC deficiency leads to chronic lung injury and increased immune cell infiltration and inflammatory responses in mice (Yu et al., 2018). Activation of AC, on the contrary, shows anti-inflammatory and anti-apoptotic effects in host cells treated with periodontal bacteria (Azuma et al., 2018). Although carmofur has shown its potential in ALI treatment, toxicity is still the primary concern. The cytotoxic effects of carmofur are mainly attributed to the release of 5-FU, its active drug in chemotherapy, which suppresses DNA synthesis by blocking thymidylate synthase and inhibits AC that hinders cell proliferation (Realini et al., 2013; Lai et al., 2017). Although the effective therapeutic dose of carmofur for attenuating ALI is 3–10 mg/kg, which is lower than the dose used in classic chemotherapy (2.85–11 mg/kg for human, or 26–100 mg/kg for mice calculated by body surface area) (Taguchi, 1980), the cytotoxic action of carmofur could not be ignored, since the inhibitory potency of carmofur is nearly 10- to 100-fold more potent on AC than on FAAH or NAAA. Repurposing anti-cancer drugs as therapeutic agents for inflammation-related diseases may not be the most ideal approach; therefore, we are still screening other approved drugs to identify potent anti-inflammatory agents. Nevertheless, our study demonstrated that carmofur significantly attenuates ALI in LPS-induced mice, and we are able to identify potent anti-inflammatory agents by drug repurposing.

## Conclusion

In summary, carmofur, an antineoplastic drug, was identified as a potent FAAH and NAAA dual inhibitor, which was further proposed as a therapeutic agent for ALI. In Raw264.7 cells, carmofur effectively increased levels of PEA and OEA, reduced the mRNA expression of pro-inflammatory factors, and down-regulated signaling proteins involved in the NF-κB pathway. More importantly, carmofur significantly attenuated ALI in LPS-induced ALI mice *via* alleviating inflammation and neutrophil infiltration. The pharmacological effects of carmofur were blocked by PPARα antagonist MK886 and CB2 antagonist SR144528, indicating that carmofur attenuated ALI in a PPARα- and CB2-dependent mechanism. Taken together, current work demonstrated that carmofur is a promising therapeutic agent for ALI, and drug repurposing may help us rapidly identify effective therapeutic agents for inflammation-related diseases.

## Data Availability

All datasets generated for this study are included in the manuscript and the supplementary files.

## Ethics Statement

All animal experiments were performed in accordance with Guide and Care and Use of Laboratory Animals from National Institutes of Health (NIH) and approved by the Animal Care and Use Committees of Xiamen University in China.

## Author contributions

KW, YX, and ZP performed the experiments and collected the data; KW and YL analyzed data; YQ and YL conceived the experiments, designed the experiments, and wrote the manuscript. All authors read and approved the submitted manuscript.

## Funding

The present study was financially supported by grants from the Natural Science Foundation of China (816001312017 to KW, 81602974 to YL), the Science Technology project of Fujian Province (2017J01360 and 2018D0015 to KW, 2018J05145 to YL), and the Xiamen Science and Technology Program Project (3502Z20189025 to YX, 3502Z20172029 and 2502ZCQ20171000 to KW).

## Conflict of Interest Statement

The authors declare that the research was conducted in the absence of any commercial or financial relationships that could be construed as a potential conflict of interest.
