# Supplementary material for: A New Use for an Old Drug: Carmofur Attenuates Lipopolysaccharide (LPS)-Induced Acute Lung Injury via Inhibition of FAAH and NAAA Activities
Source: Front Pharmacol. 2019 Jul 19;10:818. doi: 10.3389/fphar.2019.00818 (PMC6659393; doi:10.3389/fphar.2019.00818)
Supplement: Supplementary file 1 [file DataSheet_1.docx]

A new use for an old drug: carmofur attenuates lipopolysaccharides (LPS) induced acute lung injury *via* inhibition of FAAH and NAAA activities

Kangni Wu, Yanghui Xiu, Pan Zhou, Yan Qiu, and Yuhang Li

**Supplemental Figures**

**
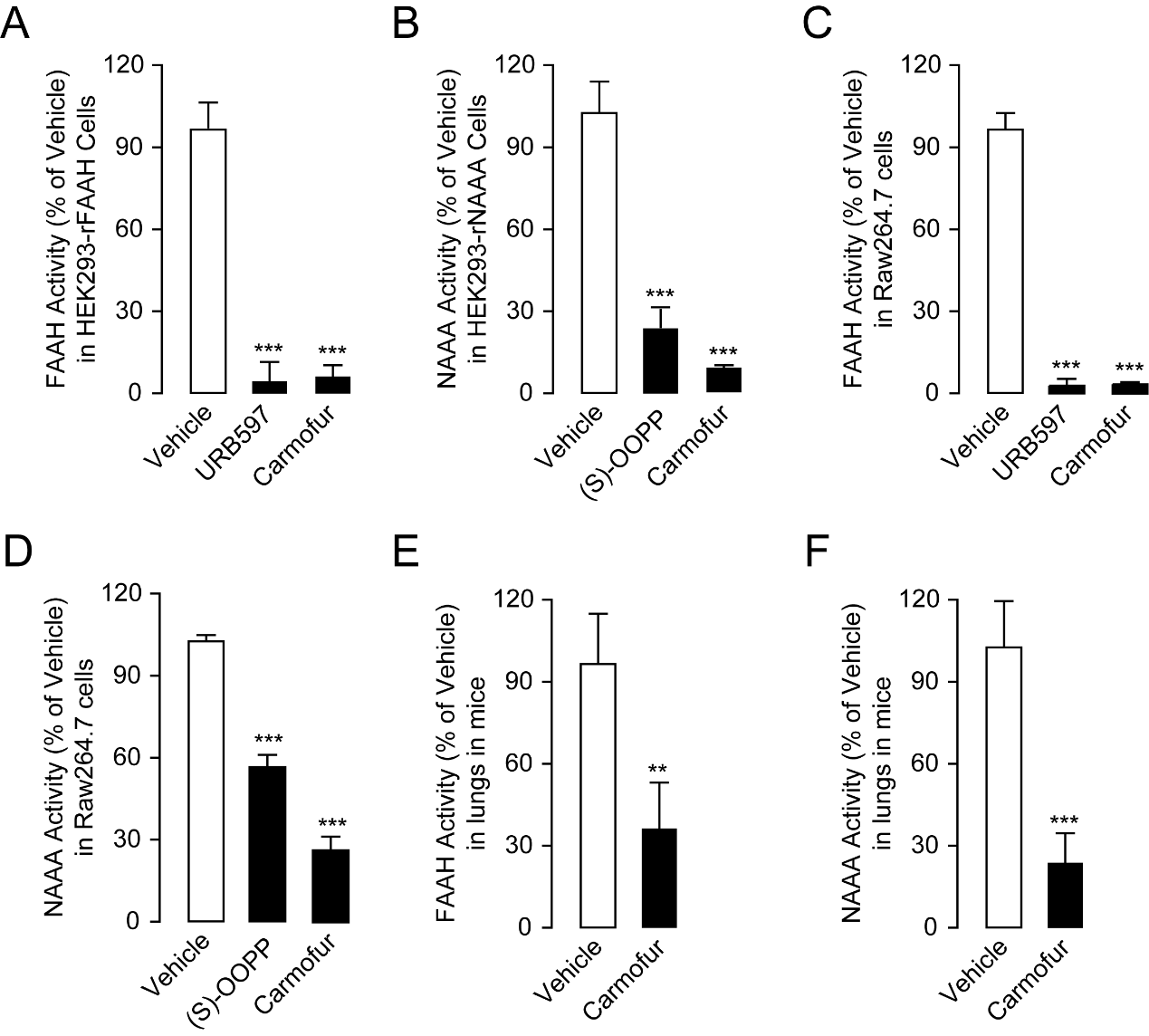
**

**Figure S1.** **Carmofur reduced FAAH and NAAA activities in Raw264.7 cells and in lungs in mice**. Inhibitory effect of carmofur (5 μM), URB597 (5 μM) and (S)-OOPP (5 μM) on FAAH and NAAA activities in HEK293-rFAAH (A), HEK293-NAAA(B) and Raw264.7 cells (C and D), n = 5. FAAH (E) and NAAA(F) activities in lungs in mice 2 h after treatment with vehicle or carmofur (10 mg/kg, orally) ***, P < 0.001 vs. Vehicle by one-way anova.


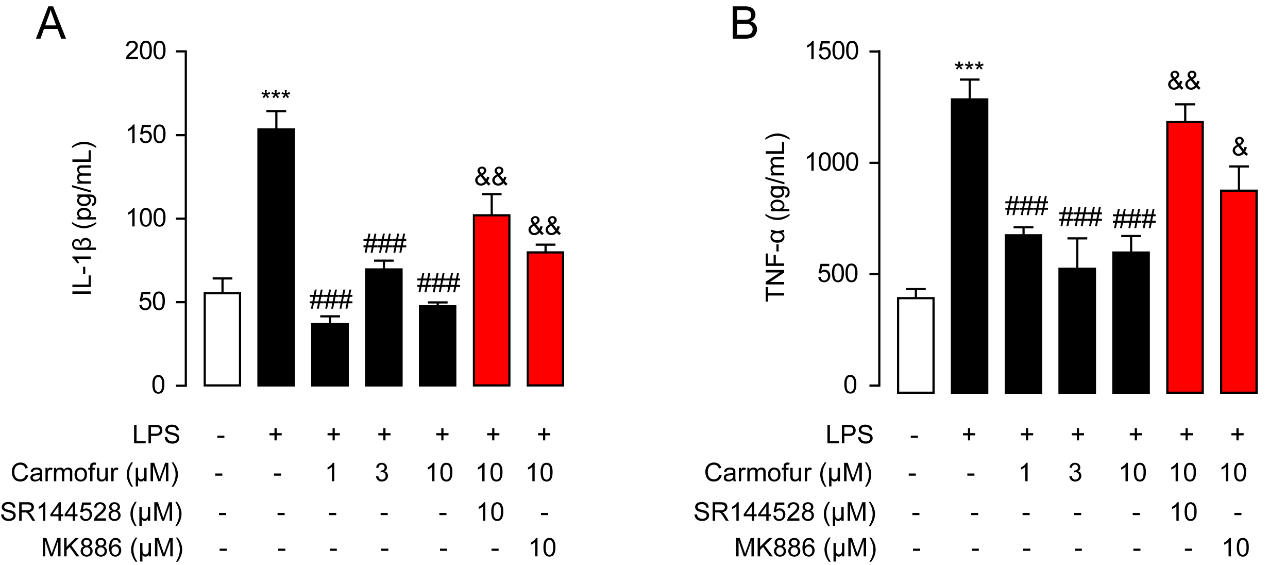


**Figure S2.** **Carmofur reduced LPS-induced inflammation in Raw264.7 cells**. Effect of carmofur, SR144528 and MK886 on protein expression of IL-1β (D) and TNF-α (G) in Raw264.7 cells treated with vehicle (0.1% DMSO) or LPS (500 ng/mL) for 72 h. Results are expressed as mean ± SEM (n = 8). **, P < 0.01; ***, P < 0.001 vs. Sham. #, P < 0.05; ##, P < 0.01; ###, P < 0.001 vs. LPS + vehicle. &, P < 0.05; &&, P < 0.01 vs. LPS + carmofur (10 μM).


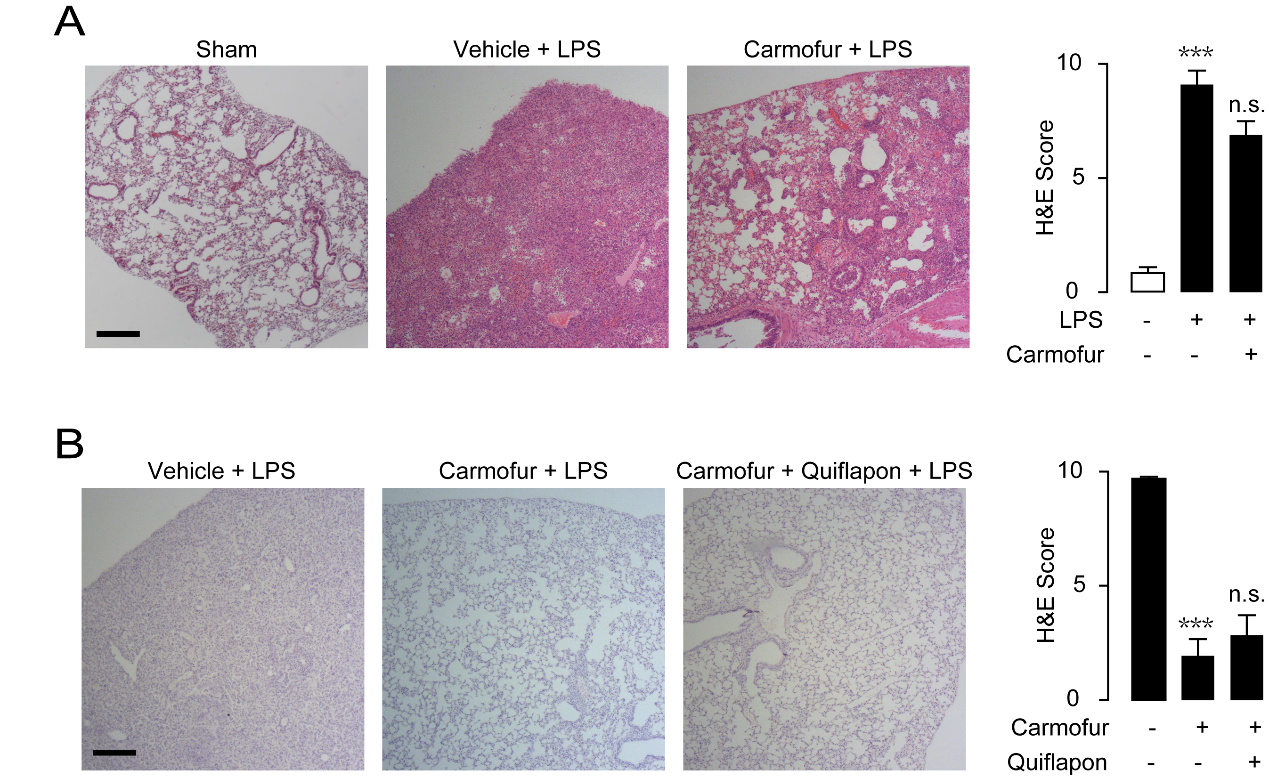


**Figure S3**. (A) H&E stained lung sections of sham PPAR-α knockout mice, and vehicle, carmofur (10 mg/kg, orally) treated ALI PPAR-α knockout mice sacrificed 3 days after LPS (5 mg/kg, i.n.). (B) H&E stained lung sections of vehicle, carmofur (10 mg/kg, orally) and Quiflapon (2 mg/kg) treated ALI mice sacrificed 3 days after LPS (5 mg/kg, i.n.). The images represent at least 75% of whole sections. Scale bar, 250 μm.

**Supplemental Materials and methods**

**S1. Enzyme activity in Raw264.7 cells and lungs**

NAAA activity was tested using a previously described method with minor modifications [1]. Briefly, Raw264.7 cells or lung tissues were first homogenized in Tris buffer (50 mM, 1.5–2.5 mL, pH 7.4 containing 0.32 M sucrose) for 3 min at 0 °C, following centrifuged at 1000 × g for 10 min at 4 °C, and the resulting supernatants were further centrifuged at 12,000 × g for 30 min at 4 °C to afford the corresponding pellet (P1) and supernatant. The supernatant was centrifuged again at 100,000 × g for 30 min at 4 °C, and the resulting supernatant was used to resuspend P1 pellet. The resuspended P1 pellet was then subjected to two freeze–thaw cycles at −80 °C followed by centrifugation again at 100,000 × g for 1 h at 4 °C. Protein concentration of the supernatant containing mice NAAA was measured by BCA protein assay kit and samples were stored at −80 °C until use.

NAAA activity was measured by incubating mouse protein (20 μg) with 25 μM heptadecenoylethanolamide at 37 °C for 30 min in 0.2 mL of phosphate buffer (50 mM, pH 5.0) containing 0.1% Triton X-100 and 3mM dithiothreitol. The reactions were terminated by adding 200 μL of methanol containing heptadecanoic acid (0.5 nmol) as internal standard. The remaining substrates were then analysed by HPLC–MS/MS. The effect of the inhibitors on FAAH activity was measured using a previously described method [1]. Raw264.7 cells or lung tissues were homogenized in ice-cold Tris-HCl (50 mM, 10 vol, pH 7.4) containing 0.32 M sucrose. Protein concentration was measured by BCA Protein Assay kit (Pierce, Shanghai, China) in spectrophotometer and 100 μg of protein was used for enzymatic assay. FAAH activity was detected at 37 °C for 30 min in 50mM Tris-HCl (pH 7.4) containing 0.05% bovine serum albumin and 100 μM of [3H]-AEA. The reactions were stopped by adding 200 μL of methanol containing heptadecanoic acid as internal standard. The remaining substrates were then analyzed by HPLC–MS/MS.

**Supplemental References**

[1] M. Alhouayek, P. Bottemanne, A. Makriyannis, and G.G. Muccioli, N-acylethanolamine-hydrolyzing acid amidase and fatty acid amide hydrolase inhibition differentially affect N-acylethanolamine levels and macrophage activation. Biochim Biophys Acta Mol Cell Biol Lipids 1862 (2017) 474-484.
